# A Rare Case of Oligosecretory Multiple Myeloma With Intracranial Plasmacytoma

**DOI:** 10.7759/cureus.114596

**Published:** 2026-08-16

**Authors:** Sidbenewende Othniel Josias Kabore, Sosthene W Ouedraogo, Aznag Mohamed Amine, Siham Ahchouch, Abderrahim Raissi

**Affiliations:** 1 Hematology and Medical Oncology, Hôpital Militaire Avicenne de Marrakech, Marrakech, MAR; 2 Clinical Hematology, Hôpital Militaire Avicenne de Marrakech, Marrakech, MAR; 3 Hematology, Hôpital Militaire Avicenne de Marrakech, Marrakech, MAR

**Keywords:** cranial nerve palsy, diagnosis, intracranial plasmacytoma, multidrug-resistant organism, oligosecretory multiple myeloma, radiotherapy, serum free light chain, treatment

## Abstract

Multiple myeloma (MM) is a hematologic malignancy characterized by monoclonal plasma cell proliferation within the bone marrow. According to current International Myeloma Working Group (IMWG) criteria, true non-secretory multiple myeloma (NSMM) is defined by the absence of detectable monoclonal protein even on serum free light chain (FLC) assay, unlike oligosecretory multiple myeloma (OSMM), in which electrophoresis and immunofixation are negative but the FLC κ/λ ratio is pathological - making diagnosis particularly challenging by conventional techniques. The association of OSMM with an intracranial plasmacytoma is exceptional.

We report the case of a 70-year-old hypertensive former smoker admitted for diffuse osteoarticular pain evolving over nine months, associated with gait disturbance and complete left oculomotor palsy. Laboratory workup revealed aregenerative anemia, thrombocytopenia, hyperleukocytosis with hyperlymphocytosis, and initially normal calcium (1.73 mmol/L), which secondarily worsened to 4.31 mmol/L during hospitalization. Serum/urine electrophoresis and immunofixation were negative, but FLC assay showed a pathological κ/λ ratio of 13.37, suggestive of OSMM. Bone marrow aspiration confirmed 75% dystrophic plasma cell infiltration. Whole-body magnetic resonance imaging (MRI) showed diffuse myelomatous infiltration with vertebral compression fractures, and orbito-cerebral MRI revealed an expansile clival lesion with bone lysis, invading the sella turcica, optic chiasm, carotid canal, and left cavernous sinus (360° carotid encasement), left petrous apex, and sphenoid sinus - for which chordoma, chondrosarcoma, bone metastasis, and lymphoma were excluded in favor of an intracranial plasmacytoma, based on convergent histological, marrow, and radiological findings. The patient was classified as International Staging System (ISS) stage I; cytogenetic testing could not be performed due to technical constraints and rapid clinical deterioration, limiting prognostic stratification (Revised-ISS (R-ISS) not calculable).

Radiotherapy targeting the sphenoidal mass was initiated, combined with corticosteroid therapy and zoledronic acid for hypercalcemia; the neurological emergency and the patient's frailty led to favoring this local approach over standard systemic therapy. The clinical course was unfavorable, marked by septic shock due to a multidrug-resistant organism, leading to death before completion of radiotherapy.

This case highlights the pivotal role of FLC assay in diagnosing OSMM when conventional techniques are negative, and the need for a multidisciplinary approach in cases of intracranial involvement. Biochemical (serial FLC) and radiological (brain MRI at three months, then every three to six months) follow-up remain recommended in the literature, as does high-performance liquid chromatography (HPLC), currently under validation for minimal residual disease detection - these points, however, remain general literature-based recommendations rather than conclusions drawn from our single observation.

## Introduction

Multiple myeloma (MM) is a hematologic malignancy characterized by monoclonal plasma cell proliferation invading the hematopoietic bone marrow, most commonly producing a monoclonal immunoglobulin detectable in the blood and/or urine. The disease is generally accompanied by at least one of the criteria known as the SLiM-CRAB criteria [1]. According to current International Myeloma Working Group (IMWG) criteria, a clear distinction should be made between true non-secretory multiple myeloma (NSMM) and oligosecretory multiple myeloma (OSMM). True NSMM is defined by the absence of any detectable monoclonal protein, not only on serum protein electrophoresis and serum/urine immunofixation, but also on serum free light chain (FLC) assay, with a normal κ/λ ratio. In contrast, OSMM is characterized by negative electrophoresis and immunofixation, but with a pathological serum FLC κ/λ ratio, reflecting minimal monoclonal secretion that is undetectable by conventional techniques but unmasked by this more sensitive assay. A plasmacytoma occurs when abnormal plasma cells accumulate at a single site, forming a solitary-or occasionally multiple-tumor, typically of bony origin, though extraosseous localization is less commonly seen. OSMM associated with an intracranial plasmacytoma is a rare and severe complication of MM. Diagnostically, these forms pose a particular challenge when serum protein electrophoresis and serum/urine immunofixation are negative, making a presumptive diagnosis of myeloma particularly difficult and potentially delaying management.

This report describes a case that precisely illustrates this diagnostic difficulty: a case of OSMM with intracranial plasmacytoma, diagnosed and managed at the Avicenne Military Hospital (AMH) in Marrakech, Morocco.

## Case presentation

The patient was a 70-year-old man with a four-year history of hypertension treated with a calcium channel blocker and a 37-pack-year smoking history, having quit in 2010, with no other notable medical history. Nine months prior to admission, he developed diffuse abdominal, pelvic, and vertebral pain, gait disturbance, and complete left-sided oculomotor palsy involving the third cranial nerve (CN III). The patient was hospitalized in the Neurosurgery Department, where abdominopelvic computed tomography (CT) revealed multiple osteolytic lesions involving both the peripheral and axial skeleton. A CT-guided biopsy of the right iliac bone established the diagnosis of plasmacytoma, and the patient was subsequently transferred to the Hematology Department for further management.

On admission to hematology, the patient presented with partial left CN III palsy. Clinical examination showed a good general condition (ECOG performance status 1), a Glasgow Coma Scale score of 15, hemodynamic stability, no signs of infection, and no peripheral edema. Neurological examination confirmed partial left CN III palsy without any other neurological deficits. Abdominal examination revealed diffuse tenderness without organomegaly.

During the hospital stay, laboratory findings included normochromic normocytic aregenerative anemia (hemoglobin 10.2 g/dL), hyperleukocytosis (white blood cell count 14,200/mm³) with hyperlymphocytosis (lymphocyte count 6,010/mm³), and, later, thrombocytopenia (platelet count 34,000/mm³). Peripheral blood smear showed erythrocyte rouleaux formation and 2% myelemia. Viral serology was unremarkable.

**Figure 1:**
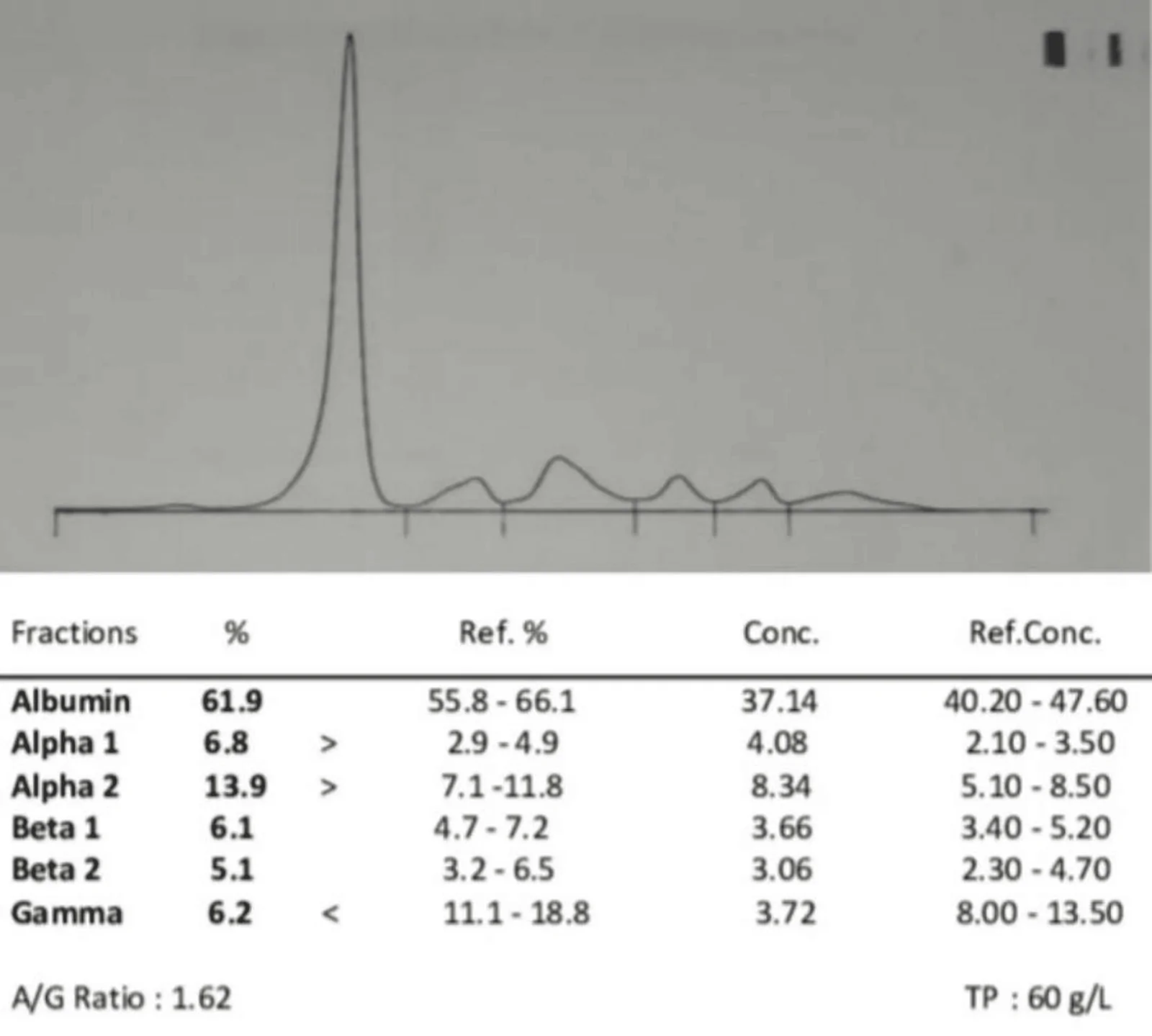
Serum protein electrophoresis Electrophoretic profile suggests hypogammaglobulinemia, to be completed by checking for Bence Jones protein in the urine.

**Figure 2:**
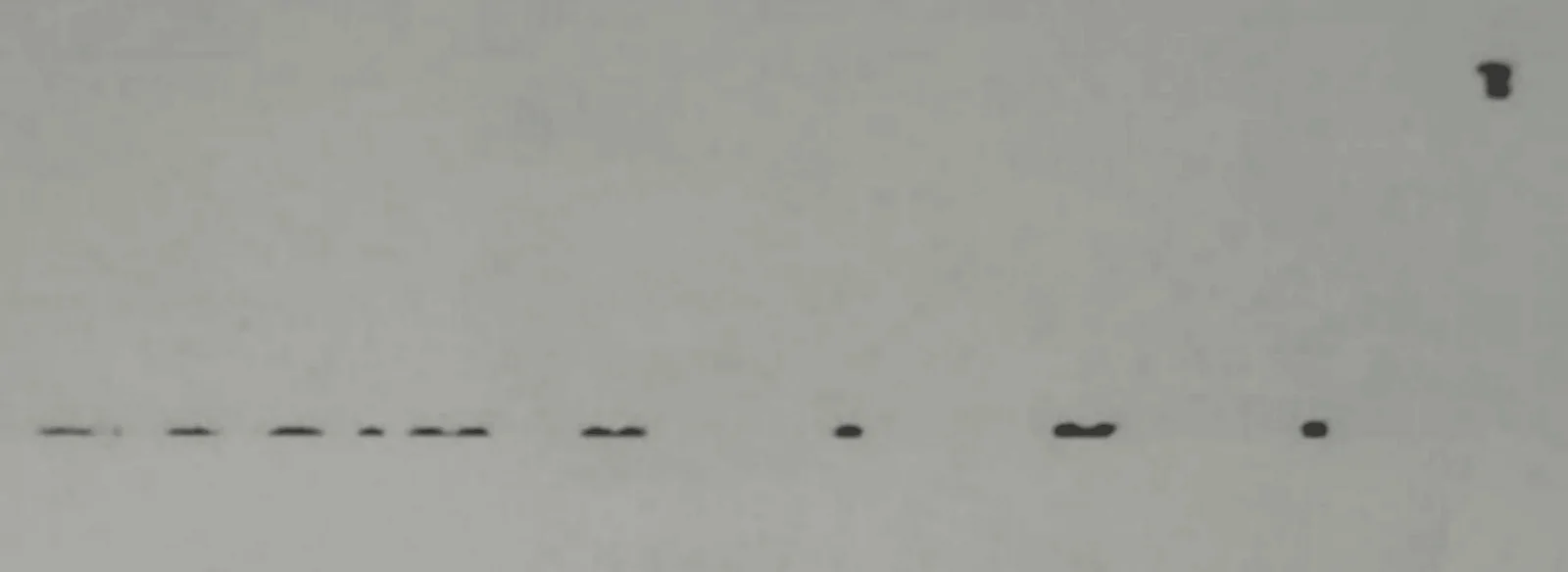
Immunofixation/Bence Jones testing No free light chains, no monoclonal component.

**Figure 3:**
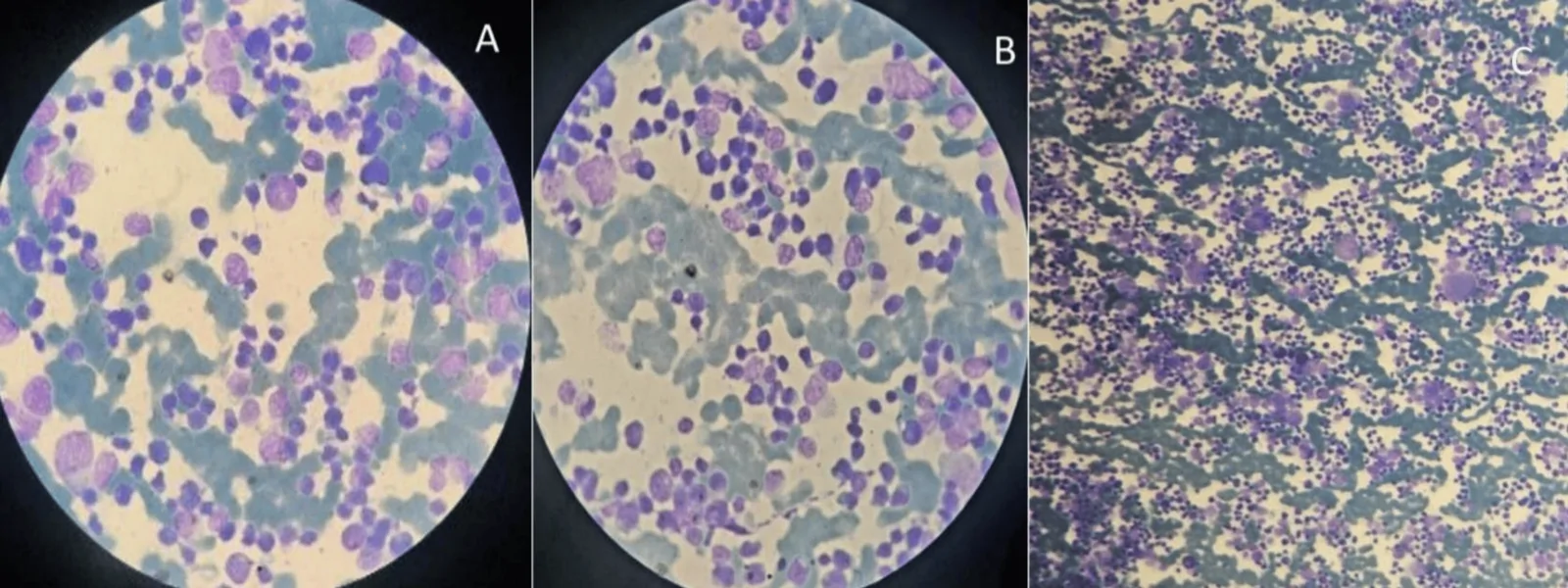
Invasion of the bone marrow by lymphoplasmacyte plasma cells (A-B) May-Grünwald-Giemsa stain, high magnification (×100) on the Olympus microscope, showing bone marrow invaded by 75% medium- to large-sized cells with basophilic cytoplasm and dystrophic morphology, suggesting plasma cells and lymphoplasmacytes. (C) May-Grünwald-Giemsa stain, low magnification (×10) on the Olympus microscope.

Serum protein electrophoresis (Figure 1) showed hypogammaglobulinemia without a monoclonal peak, confirmed by negative immunofixation/Bence Jones testing (Figure 2). Serum FLC assay demonstrated elevated kappa light chains with a pathological κ/λ ratio in the absence of renal impairment. Bone marrow aspiration revealed 75% marrow infiltration by predominantly dystrophic plasma cells (Figure 3).

The results of the biological tests are shown in Table 1 and Table 2.

**Table 1:** Initial lab work Certain values in the tables were bolded to highlight the most clinically significant abnormalities.

Biological parameter	Results	Reference values
Leukocytes	7760 /uL	4000-11000
Hemoglobin	12.5 g/dL	13-18
Mean Corpuscular Volume (MCV)	99.2 fL	78-98
Mean Corpuscular Hemoglobin Concentration (MCHC)	34.3 g/dL	31-36.5
Platelet	143000 /uL	150000-450000
Reticulocytes	25000 /uL	-
Potassium	3.4 mmol/L	3.5-4.6
Bicarbonate	18 mmol/L	22-30
Proteins	47 g/L	62-87
Uric Acid	165.5 umolL	210-420
Urea	2.5 mmol/L	2.5-7.5
Creatinine	49.6 umol/L	60-120
Albumin	35 g/L	35-50
C-reactive Protein	44.1 mg/L	<5
Lactate Dehydrogenase (LDH)	1019	135-225
Calcium	1.73 mmol/L	2.12-2.55
B2-microglobulin	2.3 mg/L	0.7-2.4 mg/L

**Table 2:** Result of the serum free light chains test Certain values in the tables were bolded to highlight the most clinically significant abnormalities.

Biological parameter	Results	Reference values
Free light kappa chains	149.35 mg/L	3.30-19.40
Free light chains of lambda	11.17 mg/L	5.71-26.30
Kappa/lambda ratio	13.370	0.260-1.650

**Figure 4:**
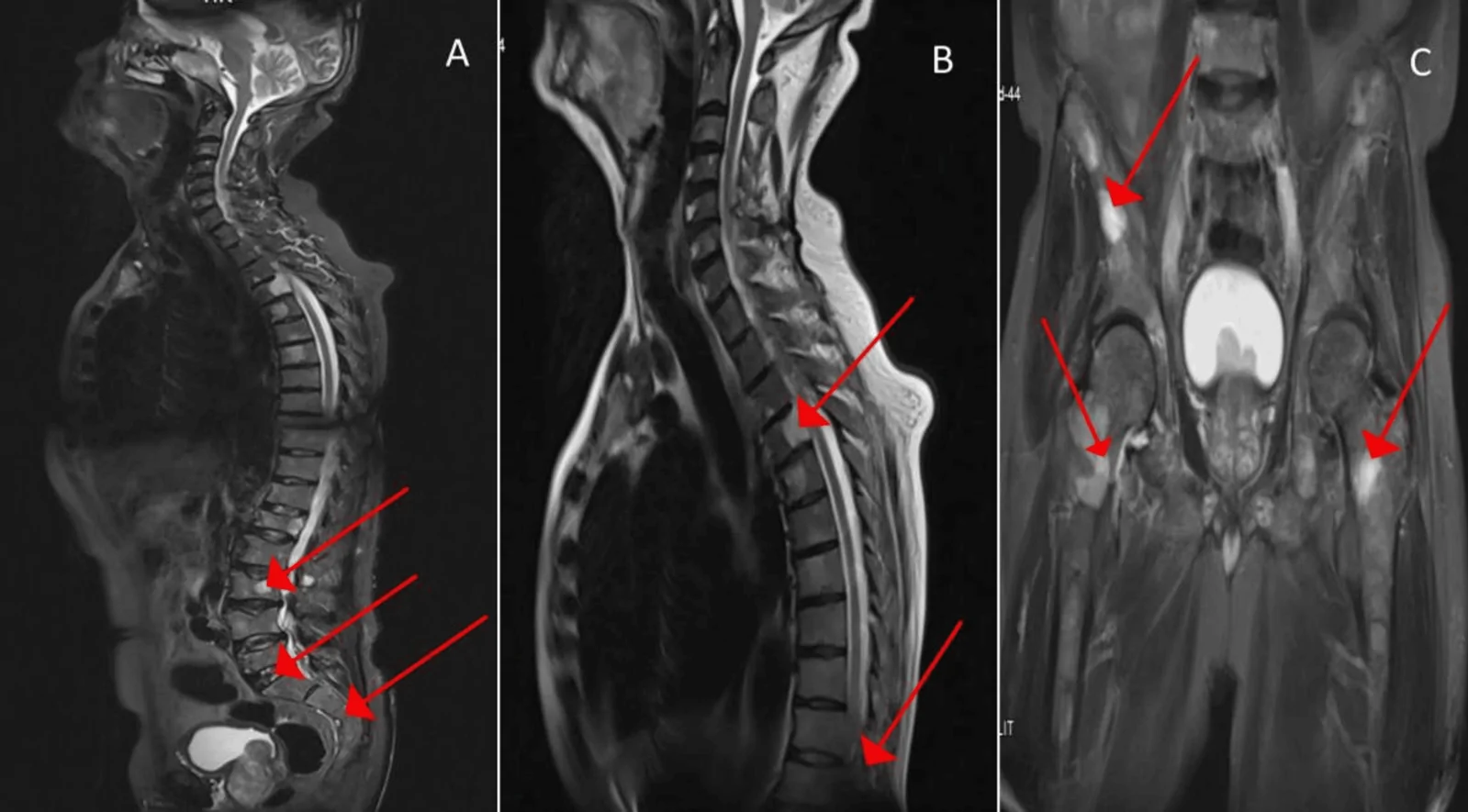
Whole-body MRI (coronal and sagittal acquisition of the spinal axis, pelvis, femurs, sternum, and shoulder girdles) Sequences weighted in T1, STIR, and DWI. Analysis according to MY-RADS criteria. Whole-body diffusion MRI shows bone marrow infiltration suggesting an active mixed-type myeloma, both focal and diffuse, according to the MY-RADS classification, with vertebral compression; an expansive process of the sphenoid bone requires follow-up orbital-brain MRI for better morphological assessment. (A-B) Nodular and patchy diffuse infiltration affecting the axial skeleton, with high T2 and STIR signal, and compression fractures of the vertebrae at D12, L1, L3, and L5 (arrows). (C) Nodular and patchy diffuse infiltration affecting the pelvis and femur, with high T2 and STIR signal (arrows). STIR: short tau inversion recovery; DWI: diffusion-weighted imaging; MRI: magnetic resonance imaging

**Figure 5:**
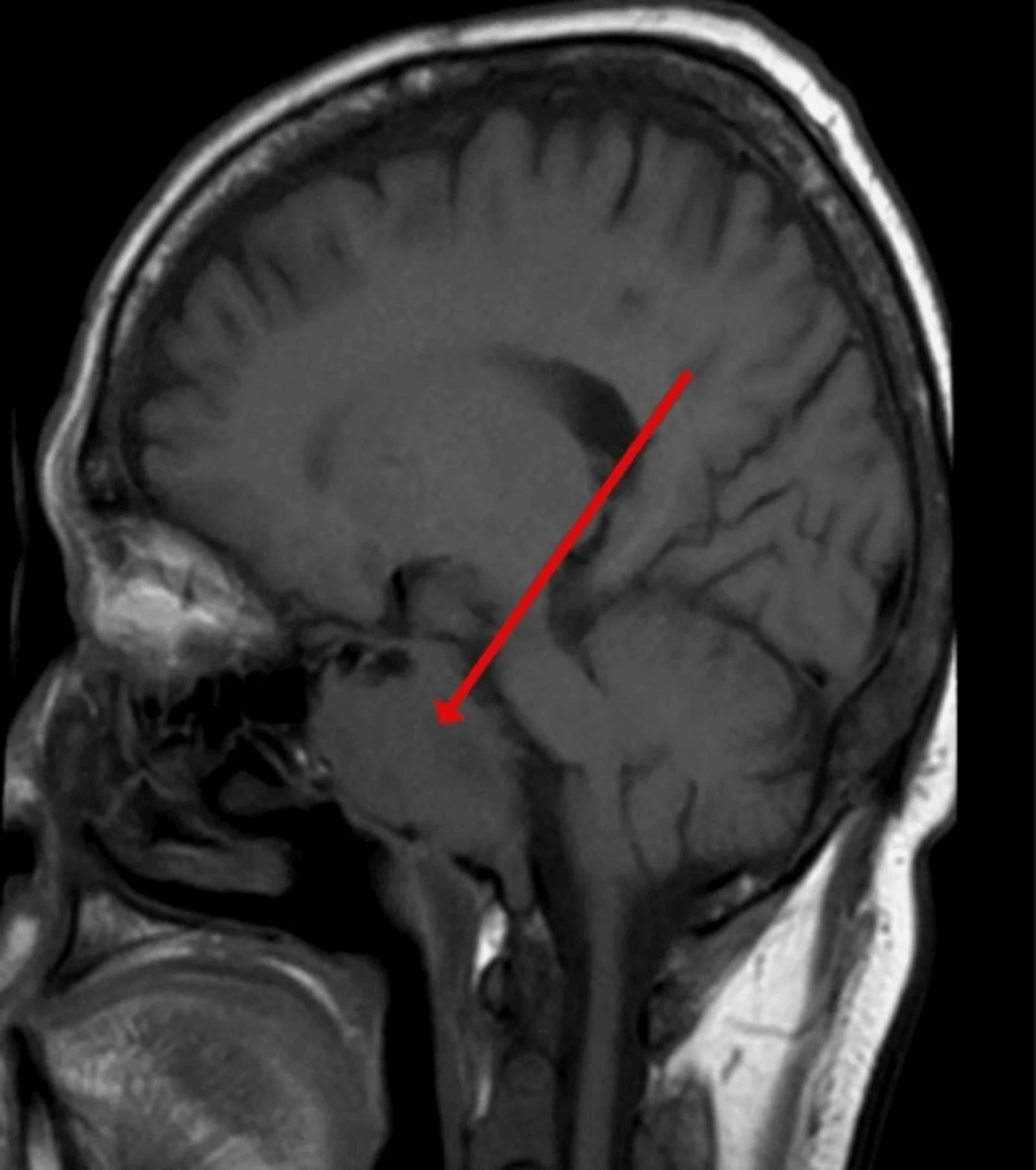
Orbital-brain MRI (clival mass with bone destruction and local spread) Expansive clival process with bone lysis and local extension, primarily suggesting a plasmacytic lesion given the context of multiple myeloma. Sagittal post-contrast T1-weighted image showing a large expansile process centered on the clivus (37.7 × 56 × 34.2 mm), isointense on T1/FLAIR, heterogeneously hyperintense on T2, without diffusion restriction, and enhancing after gadolinium injection. Local extension: superiorly, effacement of the sella turcica with probable invasion of the pituitary gland, pituitary stalk, and optic chiasm; on the left, invasion of the carotid canal and cavernous sinus, with 360° circumferential encasement of the left internal carotid artery; posteriorly, invasion of the left petrous apex; anteriorly, extension into the sphenoid sinus, more pronounced on the left. FLAIR: fluid-attenuated inversion recovery; MRI: magnetic resonance imaging

Whole-body magnetic resonance imaging (MRI) showed myelomatous bone infiltration consistent with active MM of mixed focal and diffuse pattern according to the My-RADS classification, with associated vertebral compression fractures, as well as an expansive lesion of the sphenoid bone. Complementary orbito-cerebral MRI revealed an expansive clival lesion with bone lysis and local extension, consistent with a plasmacytoma (Figures 4-5).

Given this expansile clival lesion with bone lysis, the initial differential diagnosis included chordoma, chondrosarcoma, bone metastasis (given the patient's smoking history), primary bone lymphoma, and skull base meningioma. This was set aside in favor of an intracranial plasmacytoma based on a convergence of findings: histological confirmation already established by the iliac bone biopsy, diffuse marrow plasma cell infiltration (75%), the associated multifocal osteolytic lesions, and the imaging appearance (absence of diffusion restriction, homogeneous gadolinium enhancement) consistent with a plasma cell proliferation, making an isolated primary skull base tumor unlikely.

Regarding prognosis, our patient was classified as International Staging System (ISS) stage I. Cytogenetic testing (karyotype and/or FISH for high-risk abnormalities such as del(17p), t(4;14), t(14;16)) could not be performed, owing to the unavailability of these analyses on-site and the patient's rapid clinical deterioration, marked by the onset of septic shock due to a multidrug-resistant organism. Our patient was therefore classified according to the ISS rather than the Revised-ISS (R-ISS), which incorporates cytogenetic status and lactate dehydrogenase (LDH) level, thereby limiting the precision of prognostic stratification.

During hospitalization, the clinical course was marked by general health deterioration, severe hypercalcemia (4.31 mmol/L), abdominal and lumbar pain, as well as infection with a multidrug-resistant bacillus.

Regarding treatment, the patient received radiotherapy targeting the sphenoidal mass (intracranial plasmacytoma), combined with dexamethasone and zoledronic acid for management of hypercalcemia. The clinical course was unfavorable, marked by the onset of septic shock due to a multidrug-resistant organism, leading to further deterioration and the patient's death before completion of the radiotherapy sessions.

## Discussion

NSMM was first described in 1958 by Serre and can be classified into two groups [1,2]: non-producer or producer, depending on M-protein production by plasma cells. In the producer form (85% of cases), immunoglobulins are present in the cytoplasm; these patients produce immunoglobulins but are unable to secrete them. In the non-producer form (15% of cases), no immunoglobulin production by plasma cells is observed, with the defect appearing to originate from reticular cells rather than plasma cells, seemingly blocking protein production by the latter. According to current IMWG criteria, true NSMM is defined by the absence of any detectable monoclonal protein, including on serum FLC assay, with a normal κ/λ ratio, whereas OSMM [3] is characterized by negative electrophoresis and immunofixation combined with a pathological κ/λ ratio, in the absence of renal impairment. In our case, electrophoresis and immunofixation were negative, but FLC assay revealed a pathological κ/λ ratio of 13.37 (normal range: 0.26-1.65), allowing our patient to be classified as having OSMM, in accordance with these criteria. According to the literature [4], the serum FLC ratio is abnormal in 65% of cases at diagnosis, which is consistent with our findings.

NSMM is a rare variant of classic MM. In 1%-5% of MM cases, no serum or urinary monoclonal gammopathy is detected, a condition referred to as NSMM [5]. The literature [6] reports only isolated cases of NSMM. A Moroccan study [7] involving 443 cases of MM identified five patients with NSMM (2.26%).

In our case, testing for complete or incomplete monoclonal immunoglobulins in blood and urine was negative by immunofixation, but the serum FLC assay was positive, with an abnormal kappa/lambda ratio. This more sensitive technique [8] allows accurate diagnosis of the rare cases of so-called "oligosecretory" MM. Identification of a quantifiable monoclonal component is of major importance for monitoring treatment response and long-term follow-up. Other techniques, notably high-performance liquid chromatography (HPLC) [9,10], are currently being evaluated for the diagnosis and monitoring of minimal residual disease in MM.

A study [11] on plasmacytomas and MM with intracranial extension reported an age range of 62 to 82 years at diagnosis. Our patient, aged 70 years, is consistent with this finding.

Intracranial plasmacytomas are rare lesions that can arise from the calvaria, dura mater, or skull base [12], generally following a benign course, except when associated with myeloma. A study [12] on the relationship between CD56 and CD31 expression, intracranial location, and progression to myeloma in a series of nine intracranial plasmacytomas found that, among three dural lesions, one calvarial lesion, and five skull base lesions, all five skull base lesions progressed to biopsy-confirmed myeloma in less than eight months (p < 0.05). This same study concluded that, among intracranial plasmacytomas, skull base location was the strongest factor for progression to MM. This finding is consistent with our case, in which orbito-cerebral MRI showed an expansile lesion of the sphenoid bone (located at the skull base) with bone lysis and local extension, suggestive of an intracranial plasmacytoma, given the context of MM and the immunohistochemical findings.

The clinical presentation of MM with intracranial plasmacytoma remains variable, depending on the site of compression. One reported case of MM associated with an intracranial plasmacytoma [13] initially presented with isolated, complete, and fluctuating CN III palsy. Other reported cases [14] may present with ophthalmologic involvement, among which neuro-ophthalmic manifestations are the most common (dysfunction of CNs II, III, or VI, or isolated CN VI palsy). In our case, the initial presentation was marked by complete oculomotor palsy of the left eye involving CN III, with bony signs of myeloma appearing only secondarily.

Treatment of MM with neurological involvement is not standardized. The optimal therapeutic approach for central nervous system multiple myeloma (CNS-MM) has not yet been established, and management is broadly similar to that used for lymphoproliferative disorders infiltrating the CNS, including systemic therapy, intrathecal chemotherapy, and CNS irradiation, often used in combination [15]. Most published studies emphasize the crucial role of systemic therapy in any treatment strategy. In cases presenting with signs of compression, urgent management is warranted, based on high-dose corticosteroid therapy (dexamethasone) combined with decompressive surgery (in cases of instability or severe deficit) and local radiotherapy (the standard treatment for compressive plasmacytomas) [16]. Myeloma-specific treatment consists of a triplet combining a proteasome inhibitor, an immunomodulatory drug (IMiD), and dexamethasone, or preferably a quadruplet with the addition of an anti-CD38 monoclonal antibody. Bisphosphonates are used to treat bone disease [17]. Our patient received corticosteroid therapy (dexamethasone) and bisphosphonates (zoledronic acid), and had begun radiotherapy when the clinical course became unfavorable, marked by complications (general health deterioration, multidrug-resistant bacillary infection) leading to death. The absence of cytogenetic data did not alter the initial therapeutic strategy, which was dictated by the neurological emergency related to the intracranial plasmacytoma, but could have guided the choice of systemic regimen (e.g., an upfront quadruplet including an anti-CD38 antibody in the setting of high-risk cytogenetics) had the clinical course allowed treatment to continue. This absence also represents an important limitation for prognostic interpretation: an ISS stage I classification could be falsely reassuring, whereas an unidentified high-risk cytogenetic abnormality could partly explain the aggressiveness of the clinical presentation.

The treatment monitoring strategy in OSMM with intracranial plasmacytoma: although this question remained theoretical in our case (death before completion of radiotherapy), the literature recommends serial FLC assay [18] in oligosecretory forms (target: κ/λ ratio normalization or ≥50%-90% decrease), potentially complemented by HPLC, currently under validation. Radiologically [19], brain MRI remains the reference examination, with initial follow-up at three months post-radiotherapy, then every three to six months during the first year; 18F-FDG PET-CT may help distinguish active residual disease from post-radiation changes.

## Conclusions

NSMM remains a rare hematologic malignancy whose biology is still only partially understood. Our observation illustrates how serum FLC assay allows diagnosis of OSMM despite negative electrophoresis and immunofixation, and underscores the need for multidisciplinary management when an intracranial plasmacytoma is present. The absence of cytogenetic testing, related to technical constraints and rapid clinical deterioration, limited prognostic stratification and represents a limitation of this report.

MM should be considered in any fluctuating CN III palsy, as ophthalmic signs may occasionally be the initial presenting sign. HPLC, currently under evaluation, along with standard therapeutic regimens (triplet/quadruplet therapy, radiotherapy at 4050-5040 cGy/40.5-50.4 Gy), are recommended in the literature but do not follow from our single observation, which remains illustrative rather than definitive given its nature as a single case report.
